# Portable device for presbyopia correction with optoelectronic lenses driven by pupil response

**DOI:** 10.1038/s41598-020-77465-5

**Published:** 2020-11-20

**Authors:** Juan Mompeán, Juan L. Aragón, Pablo Artal

**Affiliations:** 1 Laboratorio de Óptica, Instituto Universitario de Investigación en Óptica y Nanofísica, Universidad de Murcia, Campus de Espinardo, 30100 Murcia, Spain; 2 Departamento de Ingeniería y Tecnología de Computadores, Universidad de Murcia, Campus de Espinardo, Murcia, 30100 Spain

**Keywords:** Optoelectronic devices and components, Computer science

## Abstract

A novel portable device has been developed and built to dynamically, and automatically, correct presbyopia by means of a couple of opto-electronics lenses driven by pupil tracking. The system is completely portable providing with a high range of defocus correction up to 10 D. The glasses are controlled and powered by a smartphone. To achieve a truly real-time response, image processing algorithms have been implemented in OpenCL and ran on the GPU of the smartphone. To validate the system, different visual experiments were carried out in presbyopic subjects. Visual acuity was maintained nearly constant for a range of distances from 5 m to 20 cm.

## Introduction

Presbyopia is the reduction of the accommodation range of the human eye. Many authors have performed experiments to understand the reasons behind such reduction, which involves the lens and the ciliary muscle. Using ex-vivo eyes, Fisher^1^ and Glasser and Campbell^2^ found that the main reason for the accommodation range decrease was the elasticity loss in the eye lens, a natural process associated to aging. Heys et al*.*^3^ also found an increase in the stiffness of the nucleus of the human lens, suggesting that it could be a major factor in presbyopia development.

Presbyopia develops through a long time and varies across different people. Children can usually accommodate more than 10 D, meaning that they can focus objects being as near as 10 cm^4^. The accommodation range decreases through life until people are in their late fifties^5^. Even if the reduction in the accommodative range starts developing at early ages and it slightly increases throughout the whole life, it is not commonly noticeable for daily tasks until reaching the forties, when people start extending their arms to be able to read texts or see images. At the age of fifty, presbyopia has advanced up to the point that non-myopic people are unable to read small texts if they are held at a closer distance.

There are many available solutions for correcting presbyopia, being the most common progressive spectacles, but also multifocal contact lenses or intra-ocular lenses. Those are good solutions in general, however, with some drawbacks and limitations. Some of the aforementioned treatments are invasive such as intra-ocular lenses, refractive surgery, or even contact lenses. Others do not provide enough comfort or flexibility to the subject, as it is the case of multifocal or progressive spectacles. And all of them present trade-offs: either distance or near vision is enhanced at the expense of reducing contrast. Furthermore, only a limited range of defocus positions are available. The ideal presbyopia correction would be the recovering of the accommodation capabilities present in the young crystalline lens.

The approach introduced in this paper is non-invasive and also offers a dynamic continuous focus range solution as in the natural lens. By using tunable opto-electronics lenses, it is possible to provide a focusing range only limited by the digital-to-analog converter of the system, in addition to the maximum and minimum focal length physically achievable by the opto-electronics lenses. In our prototype, a real-time pupil tracking system running on a smartphone is used to dynamically control the optical power required in the opto-electronics lenses. Providing a smooth and comfortable visual experience to the subject requires relatively fast changes in the applied power, therefore, a heavily integrated and computationally efficient system is essential to properly drive this Dynamic Auto-Accommodation Glasses.

An initial proof-of-concept prototype was set on a 2 m^2^ optical benchtop using several mirrors, lenses and a large high-speed camera^6^. Furthermore, it was attached to a desktop PC with a high-performance GPU (Graphics Processing Unit). Contrarily, the prototype presented and evaluated in this paper is a wearable solution, i.e., a completely portable and autonomous system that can be comfortably carried by a subject, where all the image processing, computing and control is performed within a smartphone. Developing an integrated device is important to test the glasses in a realistic environment. To measure the effectiveness of the proposed Dynamic Auto-Accommodation Glasses, we have performed visual acuity tests in presbyopic subjects to measure their visual acuity improvement while wearing the glasses.

Since we proposed our first proof-of-concept system^6^, the interest for opto-electronic devices for correcting presbyopia has increased and two other works have been recently presented^7,8^. Jarosz et al*.* have described a new lens featuring a 20-mm aperture and a 3 D range^9^. They have created a spectacles frame and mounted their tunable lenses with a laser-based distance sensor to control the required power of the lenses. This design is simple but has some limitations, if the subject wearing the spectacles does not stare at front of her/him, the power applied by the system might be wrong. Contrarily, our proposal uses binocular pupil tracking to accurately determine the distance where the subject’s eyes are looking at avoiding such problem.

Padmanaban et al*.* have developed another solution using commercial parts for pupil tracking and depth sensing for the tunable lenses^10^. However, as far as we know their system is not truly a mobile solution since their pupil tracking processing (Pupil Labs GmbH, Berlin, Germany) is done on a high-end desktop PC. Another work analysing the vergence-accommodation conflict in near-eye displays have used similar components as those in this paper^11^.

The final goal is to use this device like normal glasses; therefore, we need them to become wearables. Some wearables devices have been specifically designed for tracking diabetes, sleep quality and quantity, blood pressure, heart monitoring, etc. Wearables are currently a big market and a lot of chips have been developed to build them, e.g., specific cores such as those from ARM^12^ but also small and low power displays specifically designed for wearables.

The proposed Dynamic Auto-Accommodation Glasses fit inside the wearable category, although due to their complexity, they have higher computing requirements than much simpler health-tracking and/or monitoring wearables. The higher computing needs directly affects the autonomy of the device. Therefore, an energy efficient solution is a requirement since running a highly compute-intensive algorithm such as pupil tracking in a mobile platform is challenging. We have deployed the image processing, pupil tracking and control system on a smartphone (Samsung Galaxy S7, with a Samsung Exynos 8890 processor).

The performance of the system is critical for a fluent user experience since changes in accommodation occur relatively fast under natural conditions. The Samsung Exynos 8890 processor has achieved 24 frames per second while processing 320 × 240-pixel images which is enough speed to provide the required smooth experience to the user. Another key part of the system are the opto-electronic lenses. We have used a couple of tunable lenses which are able to change their focal length as a function of applied voltage (Optotune, Dietikon, Switzerland). These are polymer-based lenses with a large range of optical powers (from − 10 D to + 10 D). There is a large research body around tunable lenses with different technologies being developed. Lin and Chen presented an electrically tunable focusing and polarizer free liquid crystal lens with a 6 mm aperture^13^. Hasan et al*.* were able to create a tunable lens with a large aperture of 32 mm and a range of 5.6 D^14^.

## Results

The performance of the device has been measured and the visual acuity of 8 presbyopic subjects has been tested with and without the correction applied for different distances.

A video of 250 frames under varying light conditions has been recorded using the cameras integrated in the Dynamic Auto-Accommodation Glasses at a resolution of 320 × 240 pixels. This video has been used to accurately measure the processing speed of the OpenCL implementation running on the smartphone GPU. According to the obtained results, the Samsung Galaxy S7 is capable of processing almost 24 frames per second taking 42 ms to process each frame. This is fast enough to provide a comfortable experience to the subjects. Therefore, it enables the system to generate a smooth response to the movements of the subject’ eyes.

**Figure 1:**
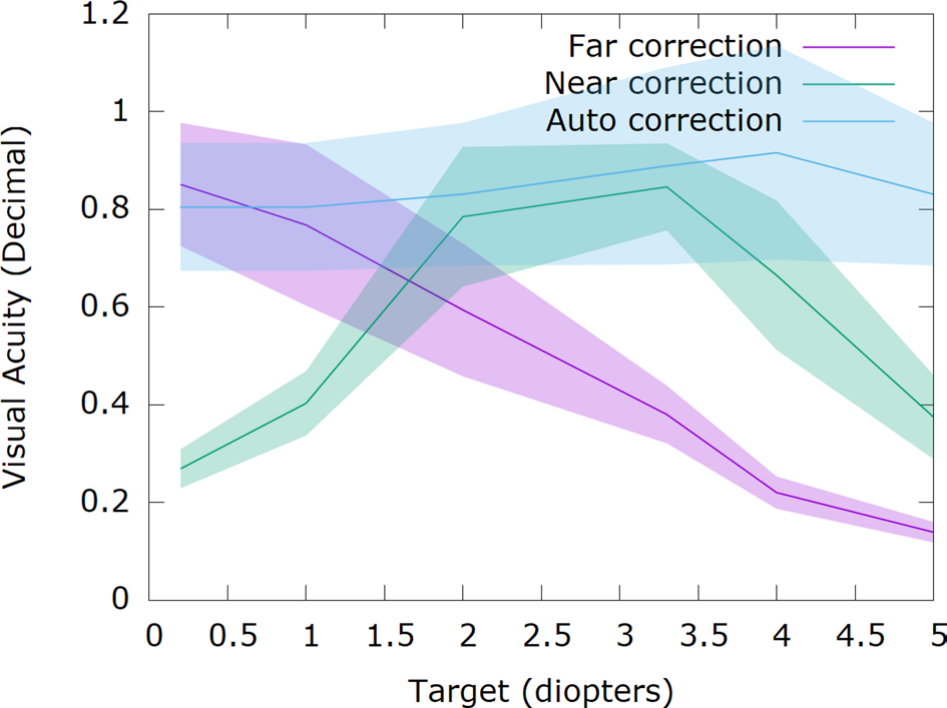
Average visual acuity in 8 subjects using 3 different corrections and tested at 6 different distances. In violet the subjects were corrected for far vision, in green the subjects were corrected for near vision (2.5 D), and in blue the subjects were using the Dynamic Auto-Accommodation Glasses. Visual acuity obtained by the subjects is shown on the y-axis in decimal scale, while the x-axis shows the distance of the objects in D.

In order to measure the effectiveness of our wearable presbyopia correction glasses, a real-life experiment has been conducted with 8 presbyopic subjects with an average age of 62.5 (minimum 52 and maximum 78) years old. The subjects were informed about the nature of the experiment and all the possible consequences. Their informed consent was collected afterwards. The purpose of the experiment is to compare three different conditions. Far correction and near correction with trial lenses, and when the subjects were wearing the opto-electronic glasses. In the far correction condition, subjects were corrected for far using their refraction value with trial lenses. This correction provides clear far vision and blurry near one. The near correction case was set by adding a near addition lens of 2.5 D. The final case was the use of the Dynamic Auto-Accommodation Glasses. Visual acuity was measured at six different distances for each one of the three corrections. This experiment was performed with the subjects standing in front of the tests. Furthermore, the tests were always placed at the same location and the subjects moved back and forth to the different distances. Even when they were wearing the Dynamic Auto-Accommodation Glasses, due to their portability there was not any limitation to accomplish the task. The selected distances were: 5 m, 1 m, 0.5 m, 0.3 m, 0.25 m, 0.2 m. Figure 1 shows the results obtained, with the horizontal axis presenting the distances where the tests were located expressed in D and the vertical axis the mean visual acuity for the three different corrections in decimal scale. The standard deviation of the visual acuity is plotted as a shadow behind each line.

With the far correction visual acuity decreases steadily as objects are closer. When the near correction was employed, subjects achieved their best visual acuity within a range of 2 to 3.3 D, with lower values for both closer and more distant targets. Visual acuity when using the Dynamic Auto-Accommodation Glasses was good for the whole range of tested distances.

A paired t-test was conducted to compare the visual acuity of the far correction and the Dynamic Auto-Accommodation Glasses when looking at 0.5 m (2 D). There was a significant difference in the visual acuity measured without the glasses (M = 0.59, SD = 0.06) and the visual acuity measured with the glasses (M = 0.78, SD = 0.14) conditions t(7) =  − 4.3658, p = 0.003291.

Furthermore, another two paired t-test were conducted to compare the visual acuity of the near correction and the Dynamic Auto-Accommodation Glasses at two distances: 5 m and 0.2 m (0.2 D and 5 D). For the first distance (5 m or 0.2 D) there was a significant difference in visual acuity measured without the glasses (M = 0.27, SD = 0.04) and with the glasses (M = 0.80, SD = 0.13) conditions t(7) =  − 9.4305, p = 0.0000314. For the second distance (0.2 m or 5 D) there was also a significant difference in visual acuity measured without the glasses (M = 0.37, SD = 0.09) and with the glasses (M = 0.83, SD = 0.15) conditions t(7) =  − 8.994, p = 0.00004284.

## Discussion

A novel Dynamic Auto-Accommodation Glasses have been developed using a mobile GPU to power and control them. This wearable device is capable of tracking the pupil of a subject in real-time, calculate his/her gaze distance and apply the right optical correction. A novel OpenCL implementation of a pupil tracking algorithm has been implemented and optimized for smartphone’s GPUs. To validate the automatic presbyopia correction glasses, an experiment has been performed testing eight subjects and obtaining a sustained improvement in visual acuity in a wide range of distances.

This device could have an impact in the life of millions of people around the world. Every person older than 50 years needs presbyopia glasses to read or look at their phones. Some presbyopic subjects only use presbyopia-correction glasses for near-distance work, however, their vision is blurred at medium or far distances and they need to remove the presbyopia glasses back and forth when changing the looking/working distances. These presbyopic subjects would be the ones that would benefit the most of a dynamic device such as the one presented in this paper. If our device would become widely available, it would change the behaviour of presbyopic subjects as they could freely look around like a (young) non-presbyopic person since everything would be automatically in focus.

The reaction speed of the device is critical to provide a smooth experience to the subject since the change in the optical power must happen very quickly yet smoothly. If the changes in the lens’ power were slow the subject would become stressed due to the reaction latency. On the other hand, if the changes were too abrupt the experience would be uncomfortable as well. Therefore, a smooth and fast response of the device to changes in the optical power required by subjects is critical. Our device fulfils those two requirements, providing a quick response under 200 ms and a smooth change in the optical power of the lenses at controlled speed of 50 D/s. This is close to the maximum speed found in children (33 D/s)^15^. Though the speed of accommodation quickly decreases with age and the maximum they measured on adults was 18 D/s.

An advantage of our device over other previously proposed^10^ is the bigger power range of the tunable lenses used. A larger range allows to focus very close targets, going beyond the normal focus range of an adult.

However, our device has some limitations as it is right now. The field of view is relatively small due to the limited size of the aperture in the used opto-electronic lenses. In particular, the field of view is 43.6° for each eye, however, due to the portable nature of the device, the subjects can move their head and look at different positions. The binocular field of view ranges from 53.1° at 0.3 m to 43.9° at 10 m. The device is still a bit bulky to be comfortable for prolonged use, and the current battery capacity does not allow for an 8-h period of continuous utilization. Fortunately, most of these problems are addressable with an improved design, although some still pose some technological challenges, i.e., the limited aperture of the lenses. However, it is reasonable to expect that larger opto-electronic lenses will be developed in the upcoming years.

From the user’s perspective the device provides a good visual experience that may require some adaptation. In the case of large changes in optical power, there are some visual effect due to the change in magnification. In addition, when the control eventually fails, some unwanted abrupt changes in the applied optical power can induce oscillations. A proper control procedure has minimized all these unwanted effects.

## Methods

The proposed Dynamic Auto-Accommodation Glasses comprise a set of parts which have to be integrated within a small and light frame in order to be portable and comfortable. This poses a number of design challenges which increases the difficulty of the setup, especially regarding the integration of the cameras, which have to be placed very close to the eye. To fulfil the wearable requirements, the smallest possible parts have been used whenever possible and a customized 3D frame has been created to properly integrate all of them. A customized board to hold the infrared LEDs has been created, and an opened micro-USB hub, with the wires directly soldered to gain space, has been employed as well. The two tunable lenses used are based on a shape-changing polymer. The lens is filled with an optical fluid, and the membrane consists of an elastic polymer. An actuator changes the pressure on the optical fluid dynamically, changing the shape of the membrane depending on the applied pressure. Therefore, by applying different amounts of current to the actuator the focal length of the tunable lens can be controlled.

**Figure 2:**
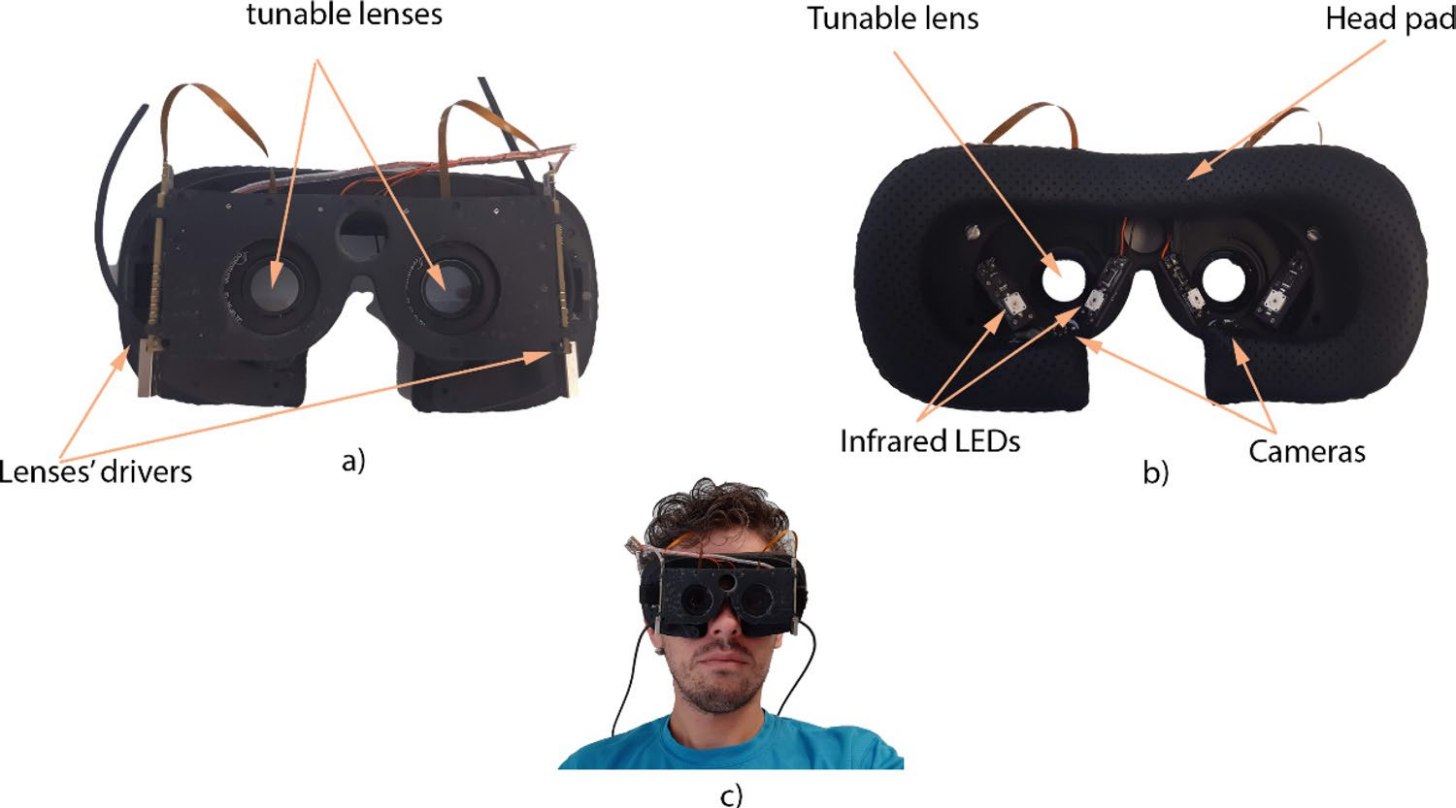
(**a**) Front view of the 3D-printed frame of the Dynamic Auto-Accommodation Glasses. The tunable lenses and their drivers are visible from this side. (**b**) Back view of the frame, the tunable lenses, the head pad, the infrared LEDs and the cameras are visible from this side. (**c**) A user wearing the Dynamic Auto-Accommodation Glasses is shown.

Figure 2a,b show a picture of the frame used for mounting the glasses. The aperture of the opto-electronic lenses is visible on both pictures. The infrared LEDs are on both sides of the lenses and the cameras just below them. Furthermore, the lenses’ drivers are on both sides of the frame. A soft pad has been used to cover the frame and to make the glasses more comfortable for the subject. Figure 2c shows a subject wearing the device.

The full list of parts mounted in the dynamic presbyopia glasses is the following:

- 2 opto-electronic tunable lenses (Optotune, model EL-16-40-TC-VIS-20D) and 2 lenses drivers.
- 2 USB infrared cameras from Misumi (Taipéi, Taiwan).
- 1 micro-USB hub with 4 USB ports.
- 4 infrared LEDs.
- 1 battery (300mAh 3.7 V) to provide power to the infrared LEDs.
- Samsung Galaxy S7 smartphone, utilized as the computing device and controller.

The cameras are compatible with the UVC standard (USB Video Class) which is not natively compatible with Android, so a library has been used to drive them: *UVCCamera*^16^. The tunable lenses are controlled through the COM port. In order to connect and communicate with the COM port in Android, the *UsbSerial* library was used.

### Procedure for glasses control and calibration

The overall process works as follows. The cameras send the recorded images directly to the smartphone where they are processed. After the subject pupils have been detected (through the real-time pupil tracking algorithm) the subject’s gaze is calculated (as described later). The lens optical power to be used, depending on how far the subject is looking at, is driven by the smartphone to the opto-electronic lenses. All the required parameters are set on a user-friendly Android application that we have developed for this purpose and there are no additional connections with any other device.

There is a one-time initial calibration procedure where the subject is asked to look at a far point and the position of his/her pupils is recorded. This calibration step is almost instant (< 0.2 s) since only a few frames are needed for the calculation. As soon as the calibration is complete, the system is activated, and the opto-electronic lenses start to apply the calculated optical power. The smartphone is continuously grabbing frames from the cameras, therefore, if the previous frame is still being processed the current frame is discarded. After each image is processed the gaze of the subject is recalculated and the new optical power is applied by the opto-electronic lenses.

**Figure 3:**
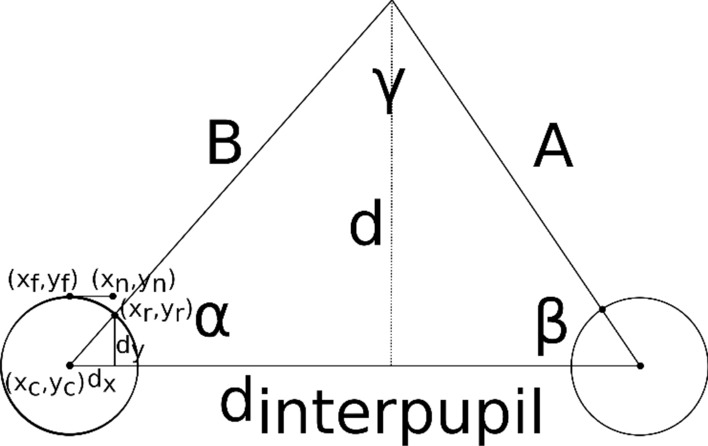
Scheme of the vergence calculation. Two eyes looking at a near target. The rotation of both eyes (α and β) is used to calculate the exact distance (d) where the subject is looking at. (x_c_; y_c_) represents the center of the eye, (x_f_ ; y_f_ ) represents the center of the pupil when the subject is looking at far, (x_n_; y_n_) is the center of the pupil when the subject is looking at near, and (x_r_; y_r_) is the real center of the pupil when the subject is looking at the stimulus.

To calculate the current gaze of the subject some information from the calibration is needed and basic trigonometry is applied. First, the position of the pupil over the eye (ocular globe) is calculated. To do so the intersection between the eye and the position recorded by the camera is calculated. Figure 3 shows the data used from the eye. (x_f_, y_f_) represents the position of the pupil while looking at far during the calibration step; (x_n_, y_n_) represents the position of the pupil recorded by the camera while looking at a near target; (x_c_, y_c_) is the center of the eye; (x_r_, y_r_) represents the actual position of the pupil while looking at a particular stimulus; α is the current rotation of the eye. Finally, the real position of the pupil is calculated using the formula:

1 $$d_{y} = \sqrt {d_{x}^{2} + r^{2} }$$

and the rotation of the eye as:

2 $$\alpha = a\tan \left( {\frac{{d_{y} }}{{d_{x} }}} \right)$$

After calculating the rotation of each eye, the distance (d) where the subject is currently looking at can be calculated. Note that the interpupillary distance (d_interpupil_), which is calculated during the initial calibration, is also needed for calculating *d* as depicted in Fig. 3.

The final equations for calculating the distance to the target follow. First, A and B are calculated as:

3 $$A = d_{{{\text{interpupil}}}} \times \frac{\sin \alpha }{{\sin \gamma }}$$

4 $$B = d_{{{\text{int}} erpupil}} \times \frac{\sin \beta }{{\sin \gamma }}$$

and the final distance (d) to the target is:

5 $$d = \sin \alpha \times B;\,\,\,\,{\text{or}}\,\,\,\,;d = \sin \beta \times A.$$

It is important to note that in this process of calculating the distance to the target, the task with the highest computing demands is the tracking of both pupils from the stream of images grabbed by the two cameras.

### System behaviour and response

In real situations, it is common for a subject to quickly look at different objects in different viewing planes. Such quick changes of both pupil locations are calculated by the system to apply the corresponding optical power to the lenses. However, if the two viewing planes are distant enough (e.g., for plane *P1* the applied optical power is 1 D while for plane *P2* it is needed to apply 5 D) such a big optical change will result in a discomforting experience for the subject. The reason is that human eyes do not react so abruptly. First, there is a reaction time; and second, there is a smooth transition from one optical power to the other. The average reaction time since a near target is shown and the accommodation starts is around 0.37 s^17^. Furthermore, the accommodation process has an average duration of 0.25 s for a 2-D accommodation range.

**Figure 4:**
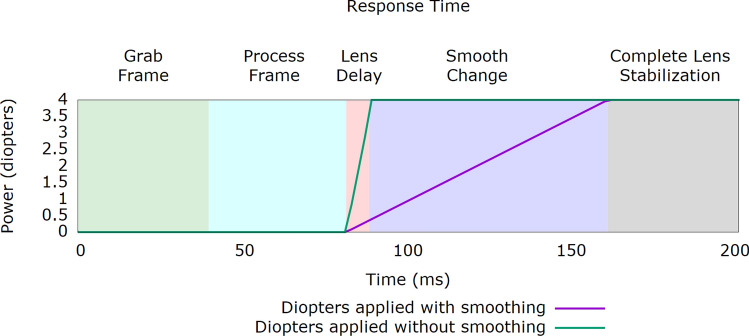
Response of the system when a change in the optical power applied to the lenses is triggered by a movement of the eyes**.** In this example, the optical power changes from 0 to 4 D over a total time of 200 ms. Two accommodation responses are shown (purple and green lines) representing the cases where a smooth transition is applied or not, respectively.

Therefore, instead of a quick and abrupt change in the power of the lenses, our presbyopia glasses have been designed to perform a smooth accommodation change as shown in Fig. 4. A speed of 50 D/s is used to change the optical power applied by the lenses. The grabbing and processing time of the frame (green and cyan areas) is included since it introduces a delay in the overall response of the system, however, this short delay is not perceived by the subject. According to the specifications of the used tunable lens, there is a 7 ms delay (pink area) before the lens reaches the final value and then it takes an additional 40 ms for the lens to completely stabilize (gray area). The purple line represents the smooth transition our system applies to the optical power of the lens to mimic the response of the eye. Finally, the green line shows how the instantaneous response would perform if no “smoothing” were applied. As mentioned before, this latter behaviour is not implemented as it is uncomfortable for the subjects.

**Figure 5:**
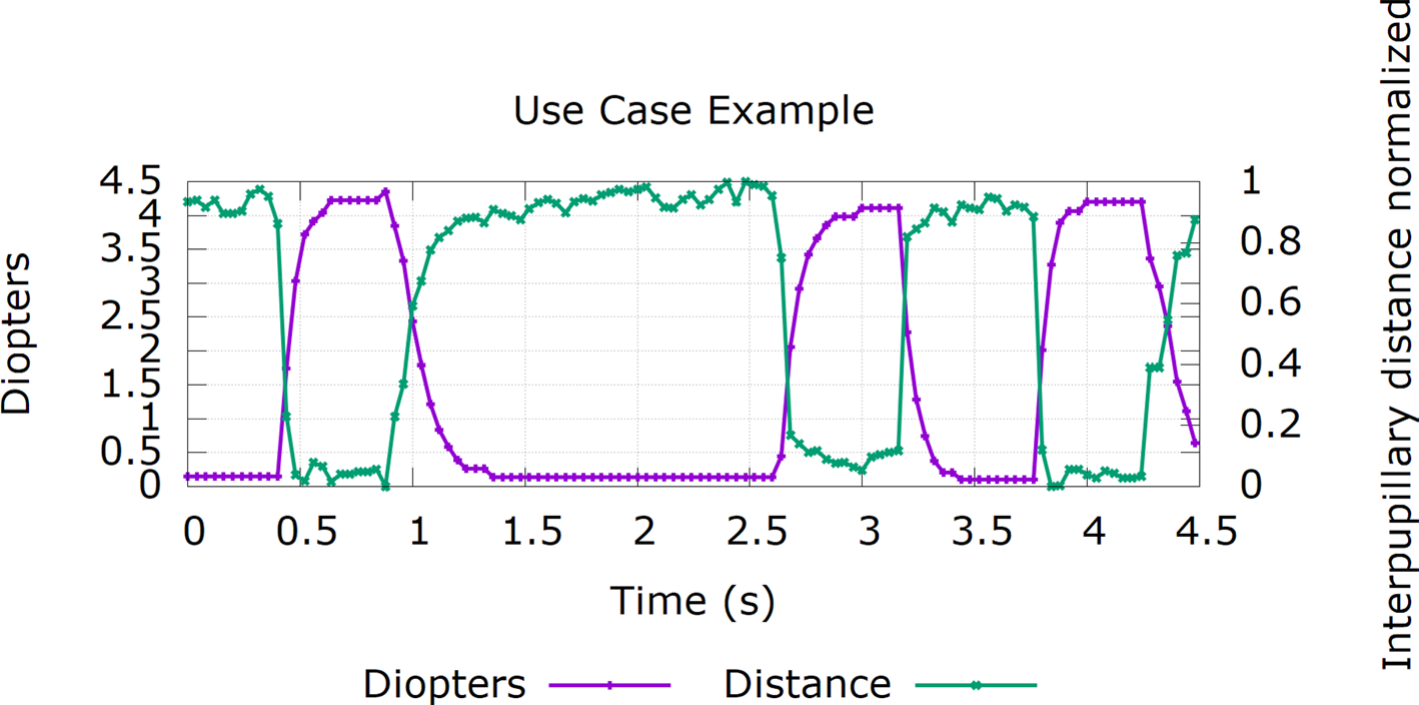
Example of the response for a subject looking at far and near alternatively. The green line shows the distance between the two pupils while the purple line represents the power applied (in D) to the lens as a response to the movement of the pupils.

To illustrate the response time and behaviour of the presbyopia glasses, an example where a subject was asked to alternatively look at a far and at a near target is presented in Fig. 5. It shows the power in D applied by the lenses (purple line) as a result of a change in the eyes’ vergence (green line). When the two eyes are closer (lower values for the *distance*) the glasses react by applying the power needed by the subject. Similarly, when the two pupils are farther away from each other (higher values for the *distance*) the applied power is reduced accordingly.

### Pupil tracking algorithm

The Starburst algorithm^18^ has been used for pupil tracking. This algorithm has been parallelized for desktop GPUs using CUDA in^19^ and later refined for binocular tracking and finer adjustment to elliptical pupils^20^. Here, we have followed a similar approach as in^20^ but instead of using CUDA, a novel implementation using OpenCL has been developed due to the incompatibility of CUDA with the used mobile computing platform. The Starburst algorithm can be divided into three main stages: a pre-processing phase to identify the corneal reflections introduced by the LEDs; a Top-hat transform is applied to the image that is thresholded. This image is dilated and the result corresponds to the detected corneal reflections. The border points of the pupil are searched as follows: a starting point is selected, which is usually the center of the image when no information about the previous pupil position is available. Then, a variable number of “rays” going from the selected center towards the limits of the image are processed. If a change in intensity bigger than a threshold is found along the ray, the point is considered a potential border of the pupil. The center of mass of the potential border points is calculated. If the distance between the current center of mass and the previous center is smaller than a threshold, the algorithm has converged, and we can proceed to the next stage. Otherwise, the search is repeated and using the current center of mass as the new pupil’s center.

Finally, when the search of the border points of the pupil has converged to a *steady* center, a RANSAC (Random Sample Consensus) ellipse fitting is applied to the complete set of border points and the best fitting ellipse is selected as the final pupil. From the previous high-level description, it can be noticed that the Starburst algorithm is highly compute demanding, since requires to process a very big amount of data.

### Mobile GPU (smartphone) implementation

**Figure 6:**
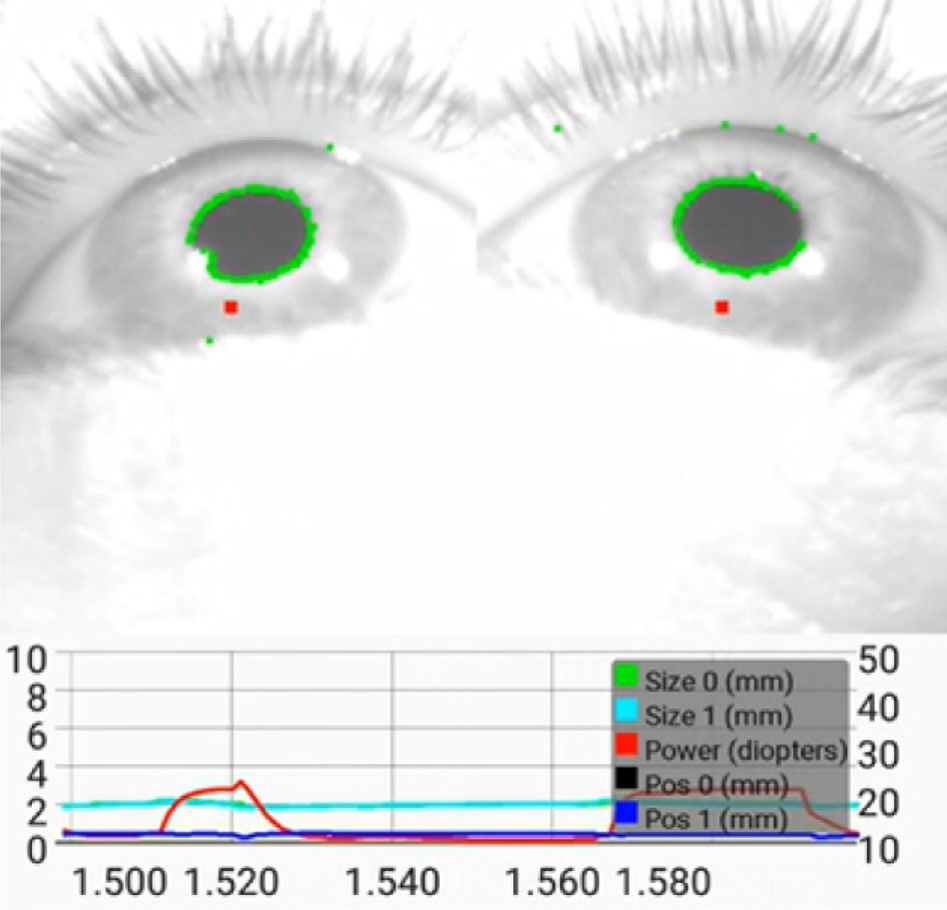
A screenshot of the control app in the smartphone. In the top both eyes with some debugging information about the pupil tracking are shown. In the bottom a plot of the pupils’ positions and size plus the power are displayed.

The mobile GPU implementation has been used for running the tests with the real subjects since it is the computing platform fully integrated in our presbyopia glasses prototype (Fig. 2), and so it was possible to easily control the cameras and the lenses. Modern smartphones are heterogeneous computing devices, including several CPU cores, a GPU and also specialized hardware for AI, video encoding/decoding, or image processing. Our implementation takes advantage of that to achieve the maximum performance from such a mobile device. A *novel* implementation of the pupil tracking algorithm has been developed from scratch by using OpenCL to take advantage of the heterogeneous architecture of mobile SoCs. The OpenCL implementation runs on the smartphone’s GPU for all the image processing and pupil tracking but the smartphone’s CPU still does a significant work managing the cameras, the opto-electronic lenses and the GUI of the Android application. The smartphone used is the Samsung Galaxy S7 running Android 7.0 which integrates a Samsung Exynos 8890 SoC, a screenshot of the controlling app is shown in Fig. 6. In order to run C++ and OpenCL in Android, the NDK kit (Native Development Kit 18b) has been used. The OpenCL implementation has been optimized to improve its performance by using vectorized memory accesses, local memory usage, native math operations (sine, cosine, etc.), kernel fusion, and reduced-precision floating point data types (half) where possible.

Vectorized memory accesses allow the GPU to pack into one load operation several pixels, therefore, increasing the efficiency of the operation and the effective bandwidth. As a result, the performance is increased due to the reduced waiting time for more pixels. Vectorized memory accesses are especially important for image processing tasks (such as the Top-hat transform, thresholding and dilation operations) which are run at the beginning of the pupil tracking algorithm. Vector data types are also used to store the location of the points found in the border of the pupil.

Local memory is capable of achieving much higher bandwidth than the device memory used to allocate the OpenCL buffers, although local memory is a much scarcer resource. Again, using local memory is useful for image processing tasks where some memory positions are accessed several times by the same threads or different ones within the same work group (warp). We have also used local memory for applying the RANSAC and for communication in the search points step. In the latter case, the local memory is dynamically reserved.

Native operations are used to calculate angles in the search of the pupil’s border points. Native math functions are optimized implementations which obtain better performance by means of reducing the precision of the operation. Some of the native operations used are: *native_sin*, *native_cos* and *native_sqrt*. The half data type is used for searching the spots and for the RANSAC step. Half data type has two advantages over float and double types: they are faster to compute due to the reduced precision and also use less bandwidth so the amount of values loaded (or stored) is potentially up to 2× or 4× more than when using floats or doubles, respectively. However, due to the reduced precision the results are less accurate. It is worth noting that the half data type has had a good support on mobile GPUs for some time, while desktop GPUs have been slower supporting this reduced-precision data type.

Kernel fusion can reduce the execution time in some cases. For instance, kernels with low execution times waste a lot of time due to the overhead of the OpenCL API calls; and also kernels which store data onto the global memory which is then loaded by the next kernel with no inter-group synchronization required. In particular, we have joined the kernels which perform the last part of the Top-hat transform and the thresholding operation, avoiding one kernel launch and saving memory operations. Finally, *separable filters* have been also used to optimize the image processing functions leading to a further reduction of the execution time.

### Ethics declaration

Every subject was informed about the requirements and aims of the study, and a written informed consent obtained, following the tenets of the Declaration of Helsinki. The ethics authorisation to perform the measurements was granted by the University of Murcia Research Ethics Committee.
