# Author Correction: Portable device for presbyopia correction with optoelectronic lenses driven by pupil response

**DOI:** 10.1038/s41598-021-86901-z

**Published:** 2021-03-26

**Authors:** Juan Mompeán, Juan L. Aragón, Pablo Artal

**Affiliations:** 1 Laboratorio de Óptica, Instituto Universitario de Investigación en Óptica y Nanofísica, Universidad de Murcia, Campus de Espinardo, 30100 Murcia, Spain; 2 Departamento de Ingeniería y Tecnología de Computadores, Universidad de Murcia, Campus de Espinardo, 30100 Murcia, Spain

Correction to: *Scientific Reports* 10.1038/s41598-020-77465-5, published online 20 November 2020

The Funding section in this Article is incomplete.

“Agencia Estatal de Investigación, Spain (Grant: PID2019-105684RB-I00 and Grant RTI2018-098156-B-C53); Fundación Séneca, Región de Murcia, spain (Grant, 19897/GERM/15).”

should read:

“Agencia Estatal de Investigación, Spain (Grant: PID2019-105684RB-I00 /AEI/10.13039/501100011033 and Grant RTI2018-098156-B-C53); Fundación Séneca, Región de Murcia, spain (Grant, 19897/GERM/15).”

